# An overview of pathophysiology and treatment options of osteonecrosis of femoral head in sickle cell disease

**DOI:** 10.15537/smj.2022.43.11.20220429

**Published:** 2022-11

**Authors:** Mohammed L. Al-Otaibi

**Affiliations:** *From theDepartment of Orthopedic Surgery, College of Medicine, King Khalid University, Abha, Kingdom of Saudi Arabia.*

**Keywords:** sickle cell disease, osteonecrosis of the femoral head, pathophysiology, treatment options, hyperbaric oxygen, cell therapies, core decompression, bone grafting, osteotomy, total hip arthroplasty

## Abstract

Osteonecrosis of the femoral head (ONFH) is the most prevalent musculoskeletal pathologic manifestation of sickle cell disease (SCD) resulting in an osteonecrotic event. This review aimed to summarize mechanisms involved in pathophysiology of ONFH and treatment options available in Saudi Arabia to treat SCD patients with complication of osteonecrotic event. The pathophysiology of ONFH include genetic and micro particles involvement. The progression of osteonecrosis involves reduced levels of bioactive compounds in peripheral blood mononuclear cells and elevated CD4+T circulating levels to stimulate pro-inflammatory cytokines contributing to inflammation at target site. Initial treatment approach is pharmacological agents use to mitigate risk. Further, bone morphogenetic protein stimulation initiate bone formation and treatment can be improved with the use of bone morphogenetic protein, total hip arthroplasty and cell therapies. This review provides baseline information for future studies to be carried out in Saudi Arabia to improve treatment options in SCD patients with ONFH.

**S**ickle cell disease (SCD) is a genetic hemoglobinopathy, inherited as an autosomal recessive trait with difficulty in hemoglobin synthesis.^1^ Sickle cell disease global burden is substantial and epidemiologically it is the most prevalent disease globally affecting approximately 2% to 17%. The birth prevalence of homozygous SCD as per global meta estimate was every 100 years (95% confidence interval (CI) = 101-123). In Europe, SCD prevalence and incidence is 0.05% to 0.01%. In Saudi Arabia, gene frequency for SCD trait is reported up to 5 per 1000 person especially in the eastern province population and SCD was reported 0.38 per 1000 person. Similarly, Sickle cell trait was reported in 4.2% of adult Saudi population and 0.26% of SCD was estimated.^2-5^

Sickle cell disease is characterized by distortion of red blood cells (RBCs) due to deoxygenation that result into both extracellular and intracellular hemolysis. This prime pathophysiological amendment intrude many other downstream effects including inflammatory cytokines production leading to micro vessels hemolysis and vaso-occlusion.^6, 7^ Sickle cell disease involve mutation in a single gene “Glu6Val” at the 6 of the beta chain gene of the pathological hemoglobin variant with an alteration of amino acid from glutamic acid to valine under deoxygenated state leading to structural changes which characterize vastly to variable clinical complications.^8^ The carrier state, sickle cell trait (homozygous form) is usually considered with the 40% presence of hemoglobin S. The chronic inflammatory state and skeletal manifestations in SCD patients are achieved by the degenerative changes in the bone marrow and bone causing perpetual production of cytokines, episodic micro-vascular occlusion, and tissue hypoxia with significantly painful episodes leading to vaso-occlusive crises (VOC) and SCD patient admission to hospital. Decrease in protein C and S (anticoagulant proteins), lipid surfaces exposure, and von Willebrand factor raised levels contribute to hypercoagulability. In 31% of SCD patients, joint and bone complications occur gradually both in acute condition such as painful VOC, acute chest syndrome or progressive chronic disability such as “avascular necrosis (AVN)” resulting in substantial morbidities including major manifestations of septic arthritis, osteonecrosis (ON), bone infarction, and osteomyelitis.^9-11^ Since SCD is considered as one of the most prevalent autosomal recessive hemoglobinopathies in the Kingdom of Saudi Arabia (KSA). Previous studies in KSA showed significant association between raised blood pressure, white blood cells count, hypoxia, and complications development in SCD (*p*<0.05).^12^ These risk factors could be controlled with better understanding of the mechanisms involved in osteonecrotic pathophysiology and better treatment options for SCD patients to improve patient’s quality of life.

This review therefore aimed to summarize mechanisms involved in pathophysiology of Osteonecrosis of the femoral head (ONFH) and treatment options currently available that could aid in the development of gold standard treatment and future clinical guidelines in Saudi Arabia to treat SCD patients with complication of osteonecrotic event.

## Osteonecrosis of the femoral head in SCD

Osteonecrosis of the femoral head or aseptic necrosis or avascular necrosis is a long term depleting musculoskeletal pathologic manifestation of SCD. It is a form of ischemic bone injury, a debilitating condition with impaired blood flow to the proximal femur bone from non-traumatic or traumatic origin with concurrent degenerative joint disease including shoulder, knee, spine or ankle. Although the exact mechanism of ON is not completely known, the suggested pathogenesis in SCD patients involve arteriolar occlusion leading to marrow edema and sinusoidal compression. This compression causes vascular stasis leading to ON. Multiple factors are involved in the onset of atraumatic ON; risk factors exposure (hemoglobinopathy, corticosteroids usage, previous trauma, Gaucher’s disease, alcohol consumption or coagulopathies) and genetic predisposition or idiopathic onset.^11,13^ The ON event begins with the impaired blood circulation to susceptible articular surfaces occurs repeatedly due to stiffness and abnormality of adherent RBCs, causing bone infarction at epiphyseal plates leading to early degenerative arthritis onset. SCD simultaneously affect multiple joints. However, femoral head is more frequently affected due to the lack of collateral blood flow making it most vulnerable to vascular insults. Irrespective of the initiating event, 3 mechanisms including extravascular compression, vascular interruption, and vascular occlusion can lead to perfusion failure and result in ischemia in femoral head through osteoclast distinction, raised homocysteine, endochondral ossification, and hypoxia as major pathways.^14^

## Prevalence of ONFH

The prevalence rate of ONFH as a major manifestation of SCD is approximately 10%.^6^ Only in Kuwait, patients with an Indian-Arab haplotype with raised fetal hemoglobin levels reported ONFH 26% in pediatrics to 50% among adults.^15^ In Saudi population, the burden of sickle cell gene and homozygous sickle cell (HbSS) related β globin haplotype in an Indian-Arab population was found to be 30% in children and 16.6% in adults.^16^ In the United States of America (USA), each year approximately 10000 to 20000 new diagnosed cases of avascular necrosis (AVN) are reported, which make 25% annual contribution to average 250000 hip arthroplasties performed yearly and overall tentative ONFH incidence in USA in early 2000s reported between 300-600 thousand new cases in general population.^17^ Literature reported 20% to 50% risk of ONFH development in patients with SCD, with 74.6% risk of ONFH as primary epiphyseal location in 60% cases during magnetic resonance imaging(MRI) scanning.^18,19^ The prevalence of SCD associated ONFH found more associated with ethnicity, profoundly reported in an African descent and overall, more prevalent among male with estimating ratio of 3 to 1.^20^

## Etiology and causative factors for ONFH

The non-traumatic etiologies of ONFH mostly include alcohol consumption and chronic usage of steroids with more than 80% contribution to disease state. Overall, the steroid associated ONFH is the second most prevailing cause after traumatic injury. Still ONFH exact pathophysiology and association with steroidal usage is unclear and considered multifactorial including endothelial dysfunction, stem cell pool of the bone marrow malfunctioning, raised intraosseous pressure, fat cell hypertrophy, and fat emboli contributing to ischemia, and consequent necrosis.^21^ The precipitation of ONFH in association with SCD involve impaired blood flow due to rigid RBCs resulting in ischemic condition and decreased oxygen supply ultimately causing bone infarction, especially at the femoral head as the most frequently targeted site of ON in SCD patients. The studies reported 20% to 50% risk during SCD for the development of ONFH.^22^

## Pathophysiology

Osteonecrosis of the femoral head is basically classified as compromised circulation of subchondral, significantly in retinacular vessels leading to bone necrosis. This aids micro fracture accumulation and cause subchondral bone to collapse because of no remodeling of bone. The clinical presentation shows a specific groin pain radiating to the knee.

The exact pathophysiology of ONFH in SCD patients is still unknown. There is a possible involvement of some genetic and micro particles in the pathogenesis of ONFH. The damage to ONFH starts in SCD patients with silent lesion in bone which progress gradually with structural bone deterioration and joints collapse leading to degenerative arthritis. The progression of ON involve significantly reduced levels of TCD4+, TCD4+ naive cells in peripheral blood mononuclear cells (PB-MNC) and elevated CD4+T circulating levels to stimulate production of produced pro-inflammatory cytokines interleukin (IL)-17+-IL4+ and IFN-γ+-IL4+ contributing to ON inflammation at target organ site. This indicate increased T helper cells production in SCD patients bone marrow and increased CD4+T subset intensify pro-inflammatory cytokines production contributing to pathological immune responses in femoral head leading to ON in SCD patients.^23^ Another factor is potential reduction in mineral to matrix ratios in necrotic cells,^24^ indicating the ratios of carbonate at 1070 cm-1, phosphate at 958 cm-1, and collagen amide I at 1070 cm-1,= respectively, through Raman spectroscopy decreasing bone mineralization and structural strength resulting in fragility fractures. Involvement of multifocal joints such as knee, shoulder, elbow and so on, along with comorbid condition such as chronic pains, VOEs, and acute chest syndrome (ACS) tend to progression of right-ONFH with decreased joint space, cysts (Steinberg stage V) and sclerosis; and minimal ﬂattening (Steinberg stage II) at the left femoral head.^25^ Further, bone morphogenetic protein (BMP) gene, a member of transforming growth factor-beta molecules superfamily, was found associated with the pathogenesis of ONFH in SCD patient. There are cytokines involved in morphogenesis and tissue development. Bone morphogenetic protein 6 polymorphisms in articular cartilage destruction during pathogenesis of ON in SCA patients showed significant association between ON and alleles G of rs267201 and A of 267196 as involved in inflammatory responses and play vital role in bone formation in association with vitamin D and para thyroid hormone (PTH).^8^ Significantly higher percent of microparticles observed in patients with ONFH indicating potential role in ON vascular pathophysiology through tissue factor (TF) and phosphatidylserine (PS) based pro-coagulant expressions presence in SCD patients, and microparticles therefore indicate presence of ONFH.^26^ The traumatic cause of ONFH include supply of blood to superolateral femoral head weight bearing segment from retinacula arteries, originating mainly from epiphyseal artery. The traumatic causes include trauma, radiation and decompression sickness. The non-traumatic pathology includes extravascular compression exposure to ischemia and intravascular coagulation.^27^

## Classification systems for ONFH diagnosis

A modified version of “Harris Hip Score” named “Children’s Hospital Oakland Hip Evaluation Scale – CHOHES” was developed and validated by Freitas et al.^28^ Freitas et al^28^ evaluate signs and symptoms and functionality status of sickle cell related ONFH. Other classification systems used for the diagnosis and staging of ONFH based on radiographs and MRI)include Steinberg or (University of Pennsylvania), Japanese Investigation Committee, Ficat and Arlet and Association Research Circulation Osseous.^29^ Osteonecrosis of the femoral head progression to femoral head collapse is primarily determined through involvement of joint and necrotic lesion volume.^30^ Another method used to predict early collapse in ONFH of hip is Kerboul method with combined necrotic angle, used in combination with MRI scans instead of radiographic images.^31^ The radiological diagnostic evaluation tools such as bone scans, MRI, computed tomography (CT) scans has made disease prognosis much more possible.

## Therapeutic approaches for ONFH

The main therapeutic options for the management of ON include non-surgical management or supportive care, surgical options including limit joint collapse such as joint preservation and replacement of joint in advance collapse.

### I. Supportive care

The natural history of ONFH secondary to SCD involves progression of degenerative alterations in hip bone. The supportive non-surgical care should be initiated at an early stage ON consisting of ambulatory assistive devices and activity modification to manage pain. The severity of the femoral head involvement determined using staging criteria with MRI scans preferably, in addition to radiographic scans. These non-operative management approaches benefit at initial disease stages before femoral head articular surface collapse.^32^ It is also recommended to transfuse packed RBCs in SCD adult and pediatric patients prior to surgery to bring preoperative hemoglobin level to 10 g/dl involving general anesthesia, as it reduces the risk of acute chest syndrome in medium risk surgeries.^33^

**Table 1:** - Demonstration of pathophysiological mechanisms involvement in osteonecrosis of the femoral head in sickle cell disease.

Author	Year	Study design	Study Population	Age	Method used	Mechanisms associated with ON pathophysiology	*P*-value
Daltro et al^23^	2020	Case–control	9 SCD patients, 15 SCD-ON patients, 19 control	10-55 year	ﬂow cytometry	CD4+T lymphocytes raised circulatory levels of simultaneously produced pro-inflammatory cytokines IL-17+-IL4+ and IFN-γ+-IL4+ contributing to ON inflammation at target organ site. The polyclonal stimulation with phorbol myristate acetate/ inomycin in vitro in SCD, SCD-ON showed significantly raised IL4+TCD4+ cells.	*p*=0.0016, *p*=0.0017
Al-Ghaithi et al^24^	2021	Experimental	7 SCD patients	22–32 years	Raman Spectroscopy	Decline in bone quality and drop in mineral volume due to decrease in bone phosphate to amide ratio, carbonate to amide ratio carbonate-to-phosphate	*p*=0.01 *p*=0.02 *p*=0.008
Adesina and Neumayr^25^	2019	Case study	1 SCD patient	19 year	-	Progression of R-ONFH with decreased joint space, cysts (Steinberg stage V) and sclerosis; and minimal ﬂattening (Steinberg stage II) at the left femoral head	*p*<0.05
Chaouch et al^8^	2015	Case–control	100 SCD patients	30±5 year	PCR/sequencing	BMP6 polymorphisms in articular cartilage destruction during pathogenesis of ON in SCA patients showed significant association between osteonecrosis and alleles G of rs267201 and A of 267196	*p*=0.04 *p*=0.0023

SCD: sickle cell disease, SCD-ON: sickle cell disease with osteonecrosis, IL: interleukin, IFN-γ: Interferon gamma, ONFH: osteonecrosis of the femoral head, PCR: polymerase chain reaction, ON: osteonecrosis, BMP: bone morphogenetic protein

### II. Surgical options

The major surgical options use for treatment of ONFH include joint preserving procedures and total hip arthroplasty.

#### A. Joint preserving procedures

i) Core decompression and variants. For early stage lesions, core decompression is considered the common treatment through reduction in intraosseous hypertension as a result of inflammatory cell infiltration and necrosis at target area.^34^ In SCD pediatric patients with ONFH use of core decompression with “bone marrow aspirate concentrate – BMAC” injection manages to alter ONFH history in skeletally immature patients. This skeletal immaturity indicates positive prognostic factor with increase chances to attain lower Tonnis grading at final check-up and return to activities without pain.^35^ A prospective study examined 10 hips with ONFH for treatment with core decompression and results showed high efficacy of core decompression in stage II and III ON on MRI.^36^ The evolution of necrosis of femoral head in SCD among 23 patients using core decompression and concentrated autologous iliac crest bone marrow aspirate injection showed significant improvement in subjective shoulder value (15%) (*p*=0.001) and simple shoulder test with 2.9 points (*p*=0.001).^37^ In 1134 hips with non-traumatic ONFH, with 80% at ON early stage patients reported total hip replacement in 38% of patients at 26 months average with core decompression without augmentation resulted in clinically improved condition in comparison to core decompression alone.^38^ Another study investigated the effects of core decompression with autologous stem cell transplantation in ONFH patients and results demonstrated drop in visual analogue score post operatively to 2.5 in 78% patients (*p*<0.0001).^39^

ii) Bone grafting. This non vascularized procedure includes remodeling and repair of the subchondral bone through necrotic bone removal, femoral head decompression, scaffolding, and structural support to femoral head.^40^ In younger patients, small to medium size lesions are treated through non vascular cortical allograft or autograft insertion to ONFH through the use of the lightbulb approach by means of anterior arthrotomy assessment through the cortical window in the femoral neck, phemister approach by means of core decompression tract assessed via lateral proximal femur and trapdoor approach using articular cartilage flap analyzed through surgical dislocation in patient seeking delay or prevention of total hip replacement.^41^

iii) Osteotomy. In younger population with low body mass index and early ONFH, especially in Asian population, proximal rotational femur osteotomy is performed among ONFH patients for necrotic lesion rotation away from weight bearing area. This can also lead to complications including hardware or nonunion failure and more challenging while converting to total hip replacement.^41^ Another type of osteotomy is angular such as valgus or varus.^40^ In cases of femoral head collapse or subchondral fracture, femoral osteotomy is the treatment of choice.^42^

#### B. Total hip arthroplasty (THA)

**Figure 1:**
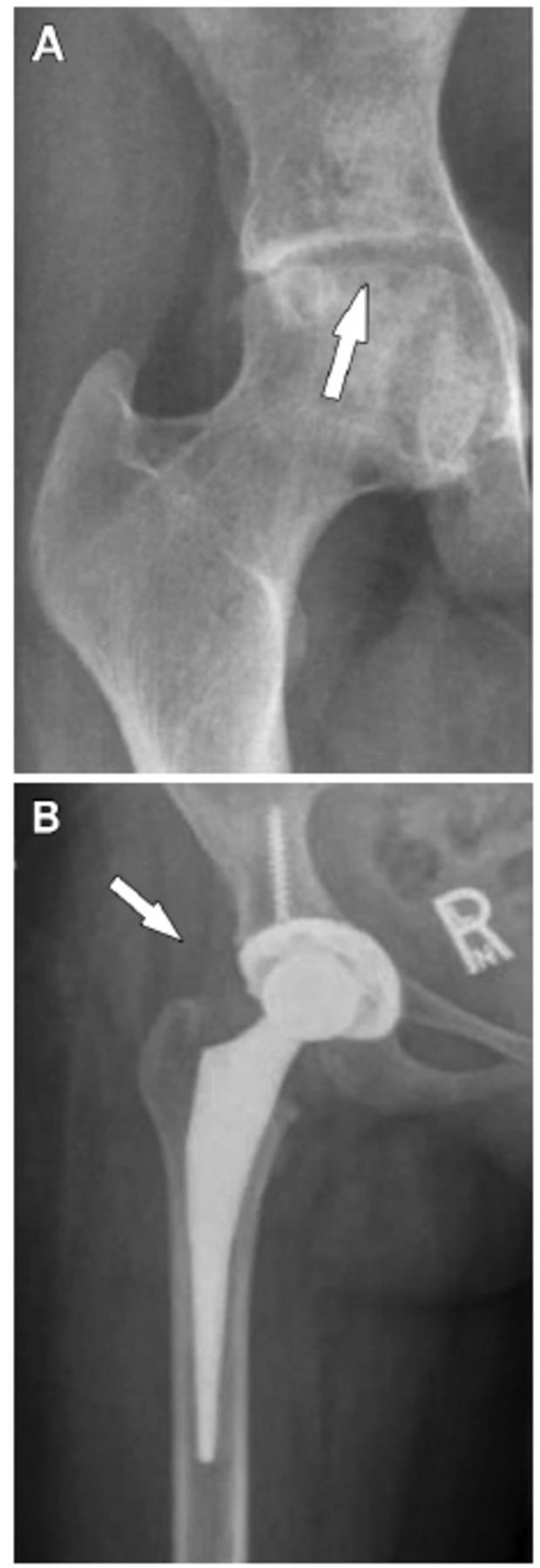
- Total hip arthroplasty. **A**) The damage due to bone flattening and osteonecrosis. **B**) A total hip arthroplasty in sickle cell disease.

The outcomes of cemented implants in SCD patients are not well established, a study^43^ reported higher revision and loosening rate of cemented implants. The cement usage usually cause thermal bone necrosis as a presentation of ON, leading to infection and loosening of cemented implant. Furthermore, the damage due to bone flattening and ON (Figure 1A) can be avoided with cement less implants during prosthetic revision surgery making procedure less dangerous for patients. This can be relieved with a total hip arthroplasty in SCD patients through decrease in pain and improved joint restoration leading to enhanced quality of life (Figure 1B). The results showed improved HSS post-operative scores and statistically significant difference between 2 groups (*p*<0.0001) at 6 months and (*p*<0.0006) after one year.^44^

### III. Other unproven therapies

i) Pharmacologic agents. Biophysical and pharmacological treatment with bisphosphates, vasodilators, anticoagulants, biophysical modalities (pulsed electromagnetic fields), and statins are under experimentation at this time. Since the ONFH pathophysiology involve intravascular occlusion mechanism, limited data is available to support the vasodilators and anticoagulants could delay disease progress.^45^ A meta-analysis reported significant improvement with bisphosphate use in epiphyseal quotients (MD: 15.32, 95% CI: 9.25-21.39) in an animal model. However, HSS score and pain scores did not show significant improvement.^46^ In early ONFH patients, bisphosphates prevent femoral head collapse through reduction in osteoclast activity through inhibition of raised turnover of bone around the necrotic region.^45^ In primary ONFH anticoagulants could be used, whereas no supportive clinical role found in secondary ONFH.^47^

ii) Hyperbaric oxygen. The inflammatory responses and cellular ischemia are supposed to be reversed with hyperbaric oxygen (HBO) in early stage symptomatic ONFH. The process in HBO involve oxygenation of hypoxic bone tissues leading to dissolved oxygen high concentration resulting in decline in edema. This result in extracellular fluid saturation along with diffused oxygen leading to ischemic bone cell oxygenation. Hence the reliance of bone cells on circulating hemoglobin decreases. A study showed positive results in statistically significant results in Asian population (*p*<0.00001) and hyperbaric chamber with 3.84 time’s higher clinical effect in comparison to control group (*p*<0.00001) (odds ratio=3.84, 95% CI: 1.87-6.64).^48^

## Novel treatment approaches

### Bone morphogenetic protein

The procedure of bone grafting with the use of BMP is used through creating a trap door at the neck and head junction of the femoral neck, causing femoral head trimming to remove necrotic bone and assure bony surface bleeding prior to proceeding with grafting.This autogenously and debridement bone grafting is associated with signaling molecules including osterix, Runt related transcription factor II (RunxII), activation protein I and β catenin. Osteoclast differentiation is primarily associated with RunxII. RunxII levels elevate by BMP stimulation through frizzled and lipoprotein receptor associated protein “LRP 5/6” receptors activation.^49^ Bone morphogenetic proteins are involved in bone formation, a study examined renal complications in SCD patients due to oxidative stress due to involvement of bone morphogenic protein receptor one (BMPR1) and significantly higher genotypes of BMPR1 (rs17022863 and rs4331783) were reported in patients (*p*=0.002 and *p*=0.008).^50^ This strengthens the use of BMPs in non-traumatic ON in early stages with core decompression and adjuvant stem cell treatment to attain better results.^51,52^

## Cell therapies

The addition of mesenchymal stem cells (MSC) to aid bone remodeling and formation in early ONFH stages could successfully maintain mitotic replication while differentiating in to various cellular types such as chondrocytes, osteoblasts and so on. The process involve the repopulation of necrotic segment bony trabeculae leading to necrotic area remodeling and regeneration.^16^ Another study^52^ used stem cell therapy in SCD patients with ONFH and reported short term good results with osteoblast injection in femur head avascular lesion as less invasive procedure bereft of unfortunate complications. Among different stem cells such as induced pluripotent, embryonic, and MSCs, in regenerative medicine MSCs have significant potential in ONFH treatment in future as most of the bioactive molecules are derived from it.^53^

## Conclusion

Osteonecrosis of the femoral head in SCD patients is a complex condition best evaluated with the use of MRI and CT scan to identify subchondral fractures through use of developed classification system. The genetic and molecular examination of patho-physiological mechanisms showed ON progression through significantly reduced levels of TCD4+, TCD4+ naïve cells in PB-MNC and elevated CD4+T circulating levels to stimulate production of produced pro-inflammatory cytokines IL-17+-IL4+ and IFN-γ+-IL4+ contributing to ON inflammation at target organ site. Bone morphogenetic protein and stem cell therapy are novel approaches and have potential to reduce associated risks and complications related to ONFH in SCD patients.

Future studies are needed to exactly know the molecular mechanism, gene editing or adjuvant cells development procedures to be explored as a safe options for treatment with growth factors and cell treatment to treat this devastating disease.
